# Added diagnostic value of SPECT to evaluate bone metastases in breast cancer patients with normal whole body bone scan

**DOI:** 10.22088/cjim.12.3.290

**Published:** 2021-04

**Authors:** Seyed Mohammad Abedi, Alireza Mardanshahi, Reza Zeanali

**Affiliations:** 1 Department of Radiology and Nuclear Medicine , Faculty of Medicine, Cardiovascular Research Center, Mazandaran University of Medical Sciences, Sari, Iran; 2 Faculty of Medicine, Mazandaran University of Medical Sciences, Sari, Iran

**Keywords:** 99mTc-MDP, SPECT, Whole body bone scan, Breast cancer, Bone metastasis

## Abstract

**Background:**

In this research, we aimed to survey the added value of single photon emission computed tomography (SPECT) in comparison with planar whole body bone scan to visualize bone metastatic lesions in patients with breast cancer.

**Methods:**

A total of 80 patients with breast cancer (invasive ductal carcinoma) were examined with planar whole body bone scan and SPECT imaging using 99mTc-labelled methylene diphosphonate (99mTc-MDP). The patients with abnormal uptakes in SPECT imaging were also investigated with magnetic resonance imaging (MRI).

**Results:**

Among these 80 patients with normal whole body bone SPECT scan, 19 (23.25%) of them revealed abnormal 99mTc-MDP uptake in skeleton. Furthermore, these 19 patients were subjected to MRI and 3 (3.75%) of them were confirmed with metastatic bone lesion.

**Conclusion:**

The obtained data suggest that SPECT possess the added diagnostic over planar whole body bone scan.

Breast cancer is one the most prominent cause of cancer-related death in females. The incidence of this cancer is independent of age, race, ethnicity, and geographic locales (1-3). Distant metastases in patients diagnosed with advanced breast cancer happens mostly in skeleton. It is reported that 30-85% of breast cancer patients will develop bone metastases. To that end, sternum, pelvis, and thoracic spine are the most susceptible site to metastases. However, the metastases involvement of other bones such as pelvis, skull and femur is also possible (4-6). It should be mentioned that bone metastases often lead to skeleton-related events such as spinal cord compression, bone fractures, pain and hypercalcemia; hence, the presence of bone metastases in patients with breast cancer heavily affects patients life quality, prognosis, and most importantly the therapy procedure (7). Due to the significant morbidity associated with breast cancer, it seems crucial to apply an effective systemic therapies to improve the survival time. To that end, early diagnosis and response assessment of possible skeletal metastases are even more prominent (8, 9). In contrast to well-known limitations of planar bone scanning such as poor specificity in staging and response assessment, it is still the main modality for staging and detection of the skeleton lesions in patients at risk of bone metastases. It is worth to note that the accuracy of bone scanning is significantly improvable with the addition of SPECT/CT (10, 11).

Bone scintigraphy using 99mTc-labelled methylene diphosphonate (99mTc-MDP) is the most common examination for visualization of the skeletal lesions including either primarily tumors or metastatic sites in malignancies like breast cancer. To that end, whole-body bone scans (WBSs) using 99mTc-MDP is the most routine test for evaluation of bone metastasis (9, 12). 

The biological distribution of 99mTc-MDP shows high uptake in the skeletal structure and urinary system. Having said that, the main downfall of using 99mTc-MDP for bone scan is lack of specificity mainly due to a known increased blood flow which is the direct result of metabolic reaction of bone to different disease processes like osteoarthritis, trauma, and inflammation (13, 14). 

The aim this study was to investigate the added value of single photon emission computed tomography (SPECT) in comparison with planar whole body bone scan for bone lesion visualization in patients with breast cancer and normal planar whole body bone scan.

## Methods

**Settings and Patients:** This study was performed in Fatemeh Zahra Hospital of Sari, North of Iran. 80 breast cancer patients referred to the hospital for whole-body bone scan (WBS) to rule out metastasis and determine staging. We excluded all the patients with abnormal whole body bone scan. 

**Whole-body bone scan:** First, a standard dose of 99mTc-MDP was injected to the patient and a planar image was acquired by a gamma camera (Siemens Company, dual headed gamma camera, E cam, Germany 2011). The patients with normal whole body bone scan were subjected to SPECT imaging. 

**Ethical Approval:** All the patients in this study agreed to contribute in this study. All the patients were informed that their health will not be endangered in the study by any means. All the patients’ information will remain confidential. This study was approved by Mazandaran University of Medical Sciences Ethics Committee. 

**Bone Scintigraphy:** First, the patients were injected with 99mTc-MDP (15 mCi). Three hours after the injection, planar whole body bone scan in the anterior and posterior positions was acquired. Scintigraphy was performed using a gamma camera (Siemens Company, dual headed gamma camera, Germany 2011). All the 64 projections were acquired at six-degree intervals. Each projection was acquired during thirty seconds, and the total time for the study was twenty minutes. For the reconstruction of SPECT images, filtered back projection was applied.

**Magnetic resonance imaging (MRI):** All the patients with positive SPECT were subjected to MRI (1.5 T whole-body MRI, Siemens). 

**Image analysis:** The planar and SPECT images were retrospectively and independently interpreted by two nuclear medicine experts which were aware of patient history of breast cancer.

**Statistical Analysis:** All the statistical analyses were performed using Prism 5.0. A p-value <0.05 is considered as a significant difference. 

## Results

**Figure 1:**
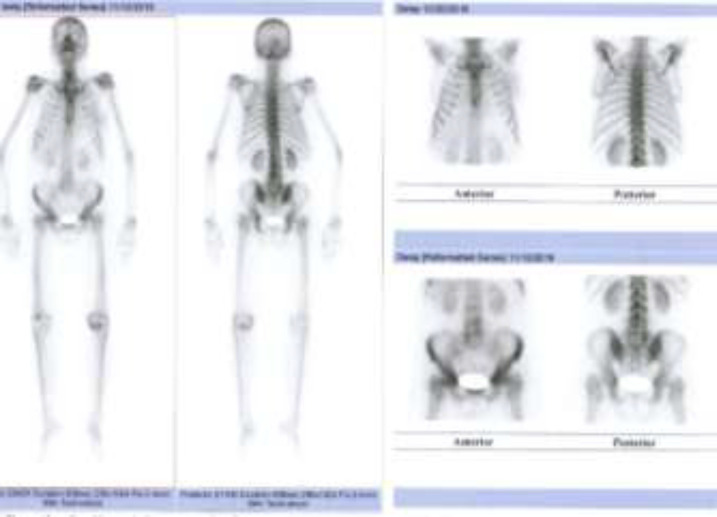
Planar whole body bone scan of a breast cancer patients

**Figure 2:**
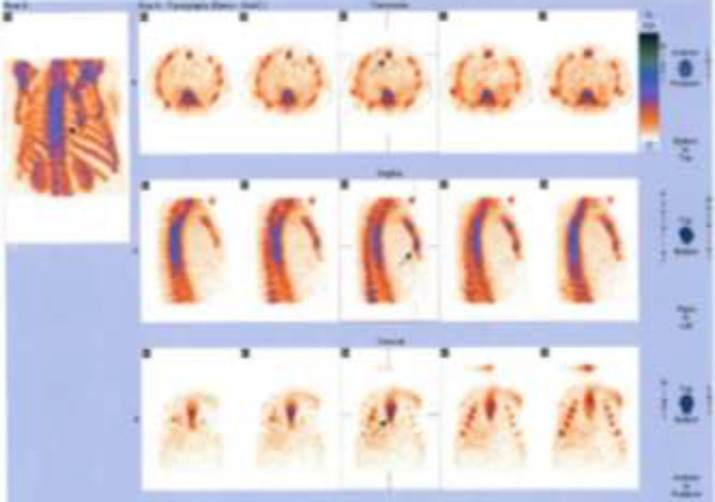
SPECT imaging of the patient displayed in figure 1. The arrow in the picture shows the metastasis site in the sternum

**Figure 3:**
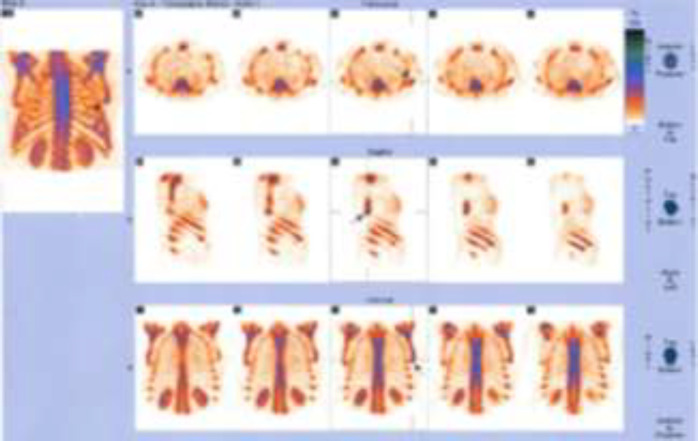
SPECT imaging of the patient displayed in figure 1. The arrow in the picture shows the metastasis site in the scapula

In this study, 80 breast cancer (invasive ductal carcinoma) patients with average age of 53.06 ± 12.74 (years) were investigated for metastatic bone lesions. First, planar whole body bone scan was obtained for every patient, then all the patients were subjected to SPECT imaging from thoracic and lumbar spine as well as pelvis. l. Among these 80 patients with normal planar whole body scan, 61 (76.75%) patients were normal and the remaining 19 (23.75%) displayed abnormal bone uptake in SPECT imaging (figures 1, 2 and 3). All these 19 patients with positive SPECT were subjected to MRI and 3 (3.75%) patients had bone metastatic involvement in their sternum, scapula, ribs and spine (table 1). 

**Table 1:** The statistics of different modalities for breast cancer patients

**Number of patients **	**Scan modality**	**Normal (%)**	**Abnormal (%)**
80	Planar bone scan	80 (100%)	0 (0%)
80	SPECT	61 (76.75)	19 (23.75%)
19	MRI	16(84.21%)	3 (15.79%)
			3.75% of 80 patients

In addition to table l data, figure 1 represents a planar bone scan which shows no abnormal radioactivity uptake in the skeleton. However, SPECT scan in figure 2 of the same patient reveals an abnormal radioactivity in the scapula and sternum. 

## Discussion

Planar whole body bone scan is a robust and sensitive imaging modality to investigate the presence of skeletal involvement in cancer patients. Unfortunately, planar whole body bone scan suffers from low specificity. Moreover, it cannot differentiate malignant skeletal lesions from benign ones. Moreover, usually the skeletal structures radioactivity uptake overlaps with accumulation of the radiopharmaceutical in various other benign situations such as infection, trauma, and degenerative changes. SPECT imaging resolves superimposition of overlying activity to a certain degree which leads to more accurate anatomical localization of skeletal lesions and aids to differentiate benign and malignant lesions. However, some lesions remain equivocal even after SPECT. 

In this study, 80 breast cancer patients with normal whole body bone scan were examined with SPECT and 19 (23.75%) of them showed abnormal 99mTc-MDP uptake. After MRI, 3 (3.75%) patients showed bone metastatic lesions. These results are in agreement with other similar studies. Recently a similar study on prostate and breast cancer bone metastasis has suggested that ambiguous lesions in 25-39% of patients were observed in planar whole body bone scan, however in SPECT images, this rate was considerably lower (15). Other studies also reported similar results (16). In another study, it is reported that planar bone scan lacks specificity while whole-body SPECT/CT results in 5.7% altered diagnosis (12 out of 212 total patients) (11). However, our study suggests 3.75% of patients (3 out of 80 patients) need change in their diagnosis while it should be considered that the number of our patents was lesser. There are numerous reports that suggest modalities such as SPECT-CT have significant impact on the final diagnosis. For example, Schillaci et al. claimed this rate of impact can be up to 40.7% (17). There are other studies which are hovering around the same results (18-20). 

In conclusion these data suggest the added diagnostic value of SPECT in comparison with planar whole body bone scan. To that end, different studies are in agreement with our study. SPECT in comparison with planar whole body bone scan improves sensitivity and specificity of bone metastases, diagnosis and significantly resolves ambiguous diagnosis in all regions of the skeleton in breast cancer patients. The diagnosis of malignant or benign bone lessons in imaging modality may have a significant influence on the management of a patient; hence SPCET is superior to planar whole body bone scan. However, it should be noticed that the investigation of a larger papulation of patients and performing MRI for all of them will lead to better results. 

## Ethical approval

The ethical standards of this study were in parallel with the Declaration of Helsinki and were approved by the local Ethics Research Committee of Mazandaran University of Medical Sciences.
